# Impact of Generation and Relocation of Defects on Optical Degradation of Multi-Quantum-Well InGaN/GaN-Based Light-Emitting Diode

**DOI:** 10.3390/mi13081266

**Published:** 2022-08-06

**Authors:** Claudia Casu, Matteo Buffolo, Alessandro Caria, Carlo De Santi, Enrico Zanoni, Gaudenzio Meneghesso, Matteo Meneghini

**Affiliations:** Department of Information Engineering, University of Padova, 35131 Padova, Italy

**Keywords:** gallium nitride, light-emitting diodes, defects, multi-quantum-well, TCAD simulations, degradation, SRH recombination

## Abstract

The defectiveness of InGaN-based quantum wells increases with low indium contents, due to the compressive strain induced by the lattice mismatch between the InGaN and GaN layers, and to the stronger incorporation of defects favored by the presence of indium. Such defects can limit the performance and the reliability of LEDs, since they can act as non-radiative recombination centers, and favor the degradation of neighboring semiconductor layers. To investigate the location of the layers mostly subjected to degradation, we designed a color-coded structure with two quantum wells having different indium contents. By leveraging on numerical simulations, we explained the experimental results in respect of the ratio between the emissions of the two main peaks as a function of current. In addition, to evaluate the mechanisms that limit the reliability of this type of LED, we performed a constant-current stress test at high temperature, during which we monitored the variation in the optical characteristics induced by degradation. By comparing experimental and simulated results, we found that degradation can be ascribed to an increment of traps in the active region. This process occurs in two different phases, with different rates for the two quantum wells. The first phase mainly occurs in the quantum well closer to the p-contact, due to an increment of defectiveness. Degradation follows an exponential trend, and saturates during the second phase, while the quantum well close to the n-side is still degrading, supporting the hypothesis of the presence of a diffusive front that is moving from the p-side towards the n-side. The stronger degradation could be related to a lowering of the injection efficiency, or an increment of SRH recombination driven by a recombination-enhanced defect generation process.

## 1. Introduction

InGaN-based quantum well light-emitting diodes (LEDs) are attracting much interest as optical sources owing to their high efficiency, small size and long-term lifetime. These devices are very versatile, since their emission spectra can be tuned from near-ultraviolet emission to red emission [1,2,3]. This can be achieved thanks to their large and tunable bandgap, by varying the composition of the ternary alloy [4]. In order to achieve long wavelengths, it is required to fabricate quantum wells with non-negligible indium contents. The drawback of this type of structure is the relatively lower internal quantum efficiency (IQE) that is related to the lattice mismatch between the InGaN QW and the GaN barrier, which induces a compressive strain that increases with indium content [5]. Internal fields and non-uniformities can also contribute. This strain causes a strong piezoelectric field that tilts the energy bands causing the separation of electron and hole wavefunctions, resulting in a reduction in the interband transition probability, and thus limiting the efficiency of radiative recombination inside the InGaN QW [6,7].

Moreover, the large lattice mismatch also favors the generation and incorporation of defects within the QW [8,9]; these defects form deep levels within the bandgap and can act as non-radiative recombination centers (NRRCs) [5,10,11], limiting the overall IQE of this type of structure.

Defects also play a key role during device degradation, because their presence can ease the generation of other defects or their relocation inside the active region, leading to an increment of Shockley–Read–Hall (SRH) recombination [11]. Defect formation may originate from a recombination-enhanced mechanism assisted by defects [12]. Finally, recent reports [13,14] suggested a new type of Auger-like recombination process, dependent on defect density, which can contribute to the lowering of device efficiency [10].

Recent studies [15,16,17] have demonstrated that the presence of an InGaN underlayer (UL) underneath the InGaN/GaN QW active region increases the IQE of InGaN QW, since it reduces the point-defect density inside the active region [18,19].

It is worth mentioning that—depending on the growth conditions—In-rich clusters may be formed within the QW. Indium spatial fluctuations are the result of a phase separation that occurs during the growth of epitaxial layers [20,21]. Phase separation represents a limitation for high indium incorporation (x > 0.3), resulting in high surface roughness and a poor crystal quality. High-resolution analysis within the In-rich regions demonstrates that In atoms tend to form clusters [22]. Similar observations have been reported for InGaN layers grown with low indium concentrations (9% < x < 20%) [22]. It has been proposed that nanoscale In-composition fluctuations due to InGaN phase separation result in the formation of In-rich clusters, which act as quantum dots (QDs) [23,24,25]. These In-rich sites have large potential energies, which strongly confine carriers and also prevent them from escaping towards the p-region. This may result in an enhancement of the radiative recombination at different photon energies compared to the surrounding semiconductor material [23].

Starting from the results of previous studies on optical degradation related to defects in InGaN devices [10], the aim of this paper was to investigate the generation and relocation of defects in InGaN-based MQW LEDs during accelerated aging. To this end, we designed a color-coded structure with two quantum wells with different emission wavelengths, in order to identify how indium composition impacts on device degradation, and to be able to identify the device region where defect generation mostly takes place. Experimental results were supported by numerical simulations in order to develop a model to describe the physical degradation processes affecting device performance. From the comparison between measurements and simulations, we analyzed the impacts that different trap concentrations and spatial locations have on the optical performance of the devices. Optical decay during aging was found to occur in two phases, with different degradation rates for the two quantum wells. We modeled degradation during the first phase with an increment of traps inside the quantum well closer to the p-side, which was assumed to be closer to the main source of NRRCs. Under this hypothesis, the presence of a diffusive front that is moving during stress causes the progressive migration of defects towards the active region. However, the optical degradation of the QW emitting at 495 nm during the second phase was found to be possibly driven by recombination-enhanced defect generation processes.

## 2. Materials and Methods

**Figure 1:**
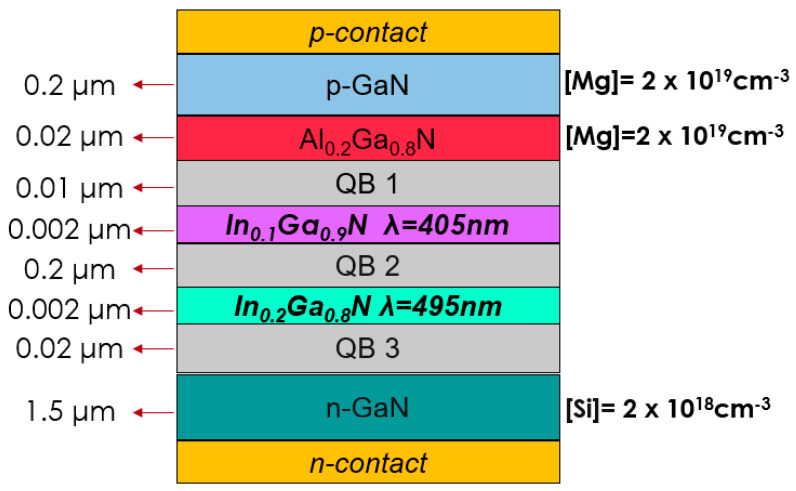
Schematic drawing of the epitaxial structure of the LEDs under test, as implemented in the simulation software.

The devices under investigation were grown by metalorganic vapor phase epitaxy (MOVPE) on a 2 inch sapphire substrate. A schematic representation of the sample structure is given in Figure 1. It consists of a 3 μm GaN buffer layer; a 1.5 μm GaN layer n-doped with silicon ([Si] = 2 × 10^18^ cm^−3^); an unintentionally intrinsically doped (u.i.d.) 20 nm quantum barrier close to the n-side, named QB3; a 2 nm quantum well with a nominal indium content of 20% and an emission wavelength of 495 nm measured at I = 100 mA (QW 495); a 20 nm quantum barrier (QB2); another 2 nm QW with a nominal indium content of 10% and an emission wavelength of 405 nm measured at I = 100 mA (QW 405); a third 10 nm quantum barrier, called QB1; a 20 nm Al_0.2_Ga_0.8_N electron-blocking layer (EBL) p-doped with magnesium ([Mg] = 2 × 10^19^ cm^−3^); and a 200 nm GaN-layer p-doped with magnesium ([Mg] = 2 × 10^19^ cm^−3^). To characterize the devices, we measured the electrical and optical characteristics. We acquired the former by means of a two-wire high-sensitive source parameter, while for the emitted optical power as a function of current (L-I) we used a photodiode. Moreover, we acquired the electroluminescence spectra as a function of current (EL-I) using a photodiode array spectrometer.

In order to study the degradation processes affecting the devices under investigation, we performed a constant-current (CC) stress test, I = 200 mA (J = 80 A/cm^2^), at high temperature, T = 350 K, for 1000 min. Temperature was controlled by a thermo-electric cooler (TEC)-based system. The stress procedure was regularly interrupted in order to monitor variations in the optical and electrical characteristics at room temperature (RT) induced by stress.

We studied the optical properties of the deep levels by means of deep-level optical spectroscopy (DLOS) [26]. The setup consisted of a xenon lamp, a monochromator, a focusing lens and an optical fiber to provide a monochromatic ray in order to excite trapped carriers in the photon range from 1.1 eV to 2.6 eV, with a step increment of 25 meV. The applied bias voltage was equal to 0 V, so that QB3 was included in the space-charge region.

The analysis of the experimental results was supported by the development of a model for the physical behavior of the device. To this end, a simulation deck was built by employing Apsys Crosslight, which is a tool for numerical simulations based on finite element analysis. The software solves the Poisson and current continuity equations in order to explain the electrical behavior. The default drift-diffusion method was modified by a non-local transport technique in order to better simulate deep quantum wells. Moreover, thermionic emission was implemented to consider transport across heterojunctions and potential barriers.

The optical characteristics of the MQW structure were computed by solving the Schrodinger equation self-consistently, by calculating the quantum confined states in a flat band condition and then applying a local correction to consider a local variation of potential. Carrier density was assumed to be confined within the well and was computed according to the local Fermi level and local confined energy states. When the potential well is under an electric field, as in our case, the well will be tilted to one side [27].

Regarding the dopant, the simulator assumes an incomplete-ionization model for the impurities. The corresponding activation energy for the Mg in the EBL is equal to 200 meV. Moreover, we considered the unintentional doping of the GaN layer by inserting n-type doping inside the active region. The impurity density is assumed to be equal to 1 × 10^16^ cm^−3^, with an activation energy in the range 24–30 meV according to the literature [28]. Regarding traps, they are considered as impurities and defined by their location, activation energy, concentration, type (acceptor or donor) and capture cross-section. Additionally, SRH recombination through traps is defined by the rate equations:

(1) $$R_{n}^{tj}=c_{nj}n\;N_{tj}\left(1-f_{tj}\right)-c_{nj}n_{1j}N_{tj}f_{tj}$$

(2) $$R_{p}^{tj}=c_{pj}p\;N_{tj}-c_{pj}p_{1j}N_{tj}\left(1-f_{tj}\right)$$

which describe the net carrier recombination rate, and where *n*_1*j*_ and *p*_1*j*_ are the electron and hole concentrations when the electron/hole quasi-Fermi-level coincides with the energy level of traps; *c_nj_* and *c_pj_* are the capture coefficients for electrons and holes, and are related to the SRH lifetime as described by the following relations:

(3) $$\frac{1}{\tau_{nj}}=c_{nj}N_{tj}\;$$

(4) $$\frac{1}{\tau_{pj}}=c_{pj}N_{tj}\;$$

(5) $$c_{n}=\sigma_{n}v_{n}\;$$

(6) $$c_{p}=\sigma_{p}v_{p}\;$$

where $v_{n}$ and $v_{p}$ are the electron and hole thermal velocities [29].

The structure shown in Figure 1 represents the model-implemented structure in the simulator. The real horizontal LED structure of the device was approximated with a vertical structure with the electrodes uniformly distributed on the top and bottom of the structure.

## 3. Results

### 3.1. Preliminary Characterization

**Figure 2:**
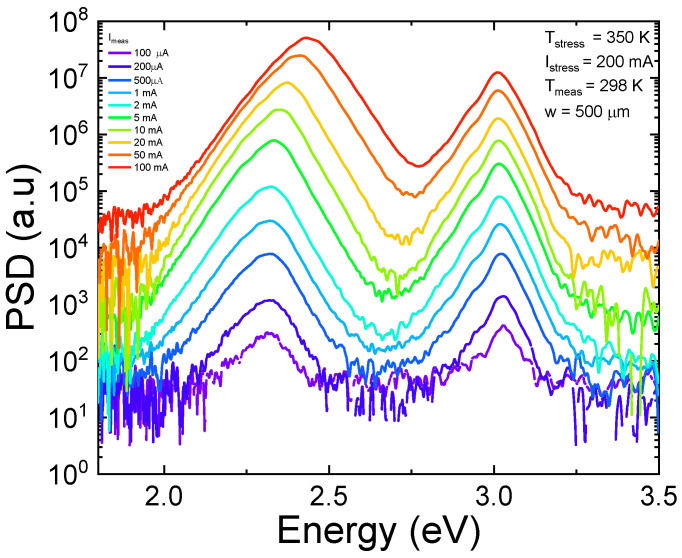
Electroluminescence spectra as a function of current measured on an unaged device. We can see that for all the injection conditions the spectra exhibit two peaks that correspond to the emissions from the two quantum wells.

**Figure 3:**
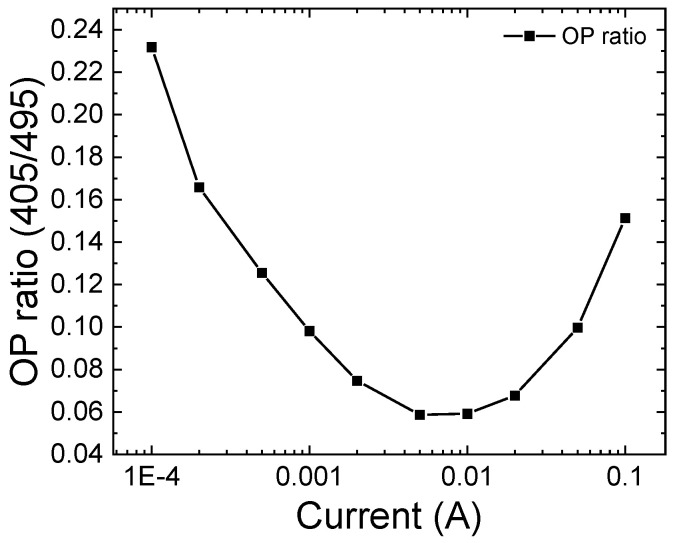
Optical power ratio calculated as the optical power emitted by the 405 nm QW divided by the 495 nm QW. The optical power ratio indicates in which quantum well radiative recombination is preferentially occurring. As the value is always below 1, emission is dominated by the 495 nm QW.

**Figure 4:**
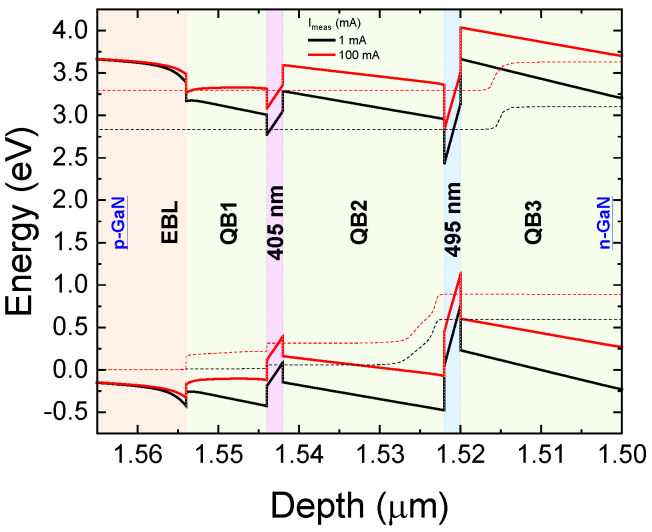
Simulated band diagram of structure for low injection level, I = 1 mA, and high injection level, I = 100 mA, at T = 300 K.

Before stress, a complete set of optical characteristics was acquired as a function of the bias current. The electroluminescence (EL) spectra measured as a function of current, here shown in Figure 2, exhibit two peaks that correspond to the emissions from the two quantum wells. Therefore, in order to evaluate in which quantum well radiative recombination preferentially occurs, we calculated the ratio between the optical power emitted by the 405 nm QW and by the 495 nm QW. As shown in Figure 3, the ratio is always less than 1, indicating that emission is always dominated by the 495 nm QW, which is closer to the n-side of the junction. Moreover, the trend in the OP ratio is non-monotonic, and the data show that emission from the 405 nm QW starts being favored again for injection currents higher than 5 mA. These results suggest that carriers are not uniformly distributed between the quantum wells, which can be ascribed to a low injection efficiency related to (i) the presence of a potential barrier in the structure or (ii) to a non-optimized doping profile [10,30]. As shown in the band diagram in Figure 4, the barrier is represented by the 20 nm QB3 and the 495 nm QW.

**Figure 5:**
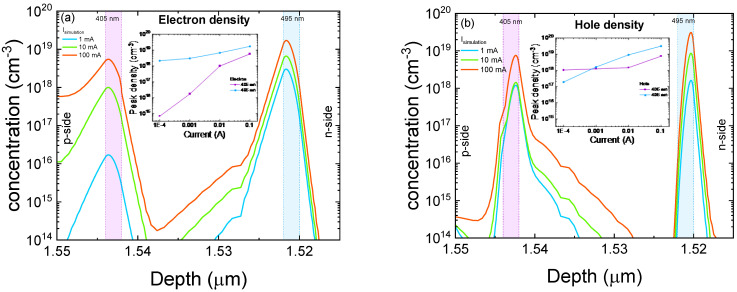
Simulated electron and hole concentrations inside the quantum wells as a function of current. (**a**) represents electron concentration, while (**b**) shows hole density.

Simulation results, shown in Figure 5a,b, confirm that for a low injection level, the 405 nm QW is lacking in electrons. As the current rises, electron injection into the 405 nm QW increases, and therefore we observe an increment of the optical power emitted by the short-wavelength well. Therefore, recombination occurs preferentially in the 495 nm QW, preventing electrons from reaching the 405 nm QW.

**Figure 6:**
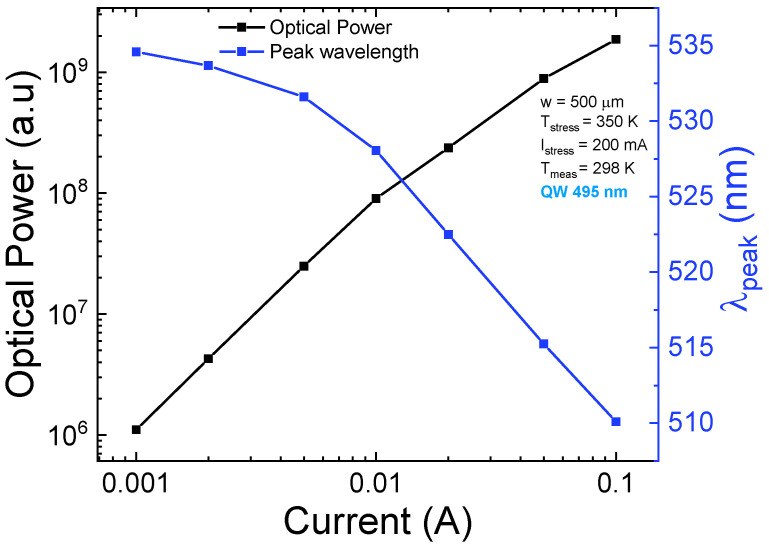
Trend as a function of the injection current of the optical power and peak-emission wavelength for the 495 nm QW. We observed a blue-shift of the wavelength, which can be ascribed to progressive band-filling and a following screening of the QCSE.

With increasing current, the increment of the optical power of the 495 nm QW is followed by a blue-shift of the peak emission wavelength (Figure 6), which can be ascribed to a phenomenon of band filling that leads to screening of the quantum confinement stark effect (QCSE) [6]. However, the 22 nm of blue-shift cannot be ascribed solely to QCSE screening and band filling; it may also be related to the presence of In-rich clusters that are filled at lower injection levels, whereas localized In-poor regions become progressively filled at higher injection levels [22,31,32]. In particular, the In-rich clusters [21] introduce local potential minima where carriers are confined instead of being separated toward the opposite sides of the well, thus enhancing the recombination efficiency [33].

However, the peak wavelength of the 405 nm QW exhibits a minor red-shift (Δλ < 1.2 nm) with increasing bias current. The screening of the QCSE in this case is strongly reduced, since carrier concentration and the internal field are lower in the 405 nm QW [4,34]. A comparison of pulsed vs. DC L-I measurements allowed us to exclude the possible role of device self-heating in the red-shift of the 405 nm QW.

#### Effect of Trap Concentration and Spatial Position on Optical Characteristics

**Figure 7:**
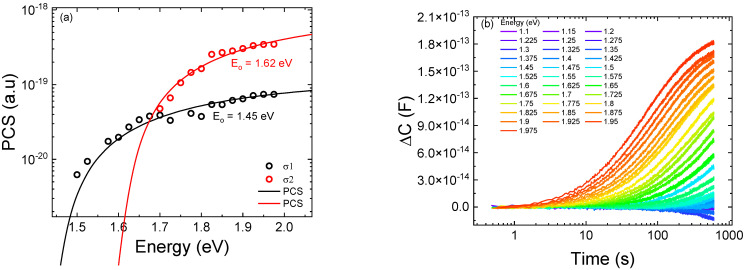
(**a**) shows the photoionization optical cross-section (PCS) derived from DLOS measurement of the sample after stress. According to the relation $E^{0}=E^{T}+D$, where *E*^0^ is the optical activation energy and D is the Frank–Condon shift, we can identify the presence of two deep levels, one of them located at the midgap, which represents the worst-case condition for SRH recombination. In (**b**), we report DLOS transient.

In order to develop a physical model able to describe the trend as a function of current of the optical power emitted by the quantum wells and the corresponding variation in the peak wavelength, we studied the effects that different trap concentrations and positions have on the optical characteristics of the simulated LED structure. We started from an ideal condition (no traps), and we selectively included trap states in each layer of the active region. For simulations, we assumed that traps are located at the midgap, in order to consider the worst-case scenario for SRH recombination [10], with a capture cross-section equal to 1 × 10^−^^15^ cm^2^. Additionally, the presence of midgap traps was confirmed by the outcome of our DLOS characterization, through which we identified two deep levels with corresponding activation energies E_t1_ = 1.45 eV and E_t2_ = 1.62 eV [26] (Figure 7).

**Figure 8:**
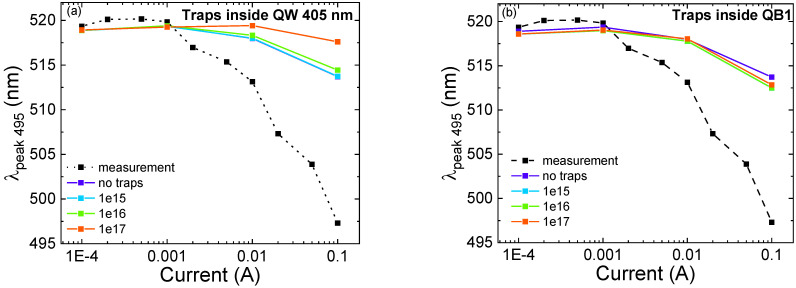
Peak wavelength variation as a function of current for the 495 nm QW. The dashed line represents the experimental results, while the colored lines are the simulation results for different trap concentrations. (**a**) shows the effect of traps located inside the 405 nm QW; (**b**) shows the effect of traps located inside QB1.

We simulated and analyzed the effect of different trap concentrations on the optical characteristics of the structure. For the static operating regime, the SRH recombination rate is influenced only by the product between *N_t_* and σ, where *N_t_* is the trap concentration and σ is the carrier capture cross-section. We kept the value of the capture cross-section constant, and we swept the values of *N_t_sim_* from 1 × 10^15^ to 1 × 10^17^ cm^−^^3^. Regarding the 495 nm QW, we partially reproduced the observed blue-shift, confirming the hypothesis that it is related to band-filling phenomena and screening of the QCSE. However, from the experimental analysis, we measured a blue-shift of 20 nm (16 meV), while the simulated blue-shift in the “no traps” condition (purple line in Figure 8a,b) is equal to 5 nm (4 meV). As previously mentioned, the stronger blue-shift observed in the simulations can be ascribed to the presence of localized indium clusters, which are not considered by the simulator [32,33].

**Figure 9:**
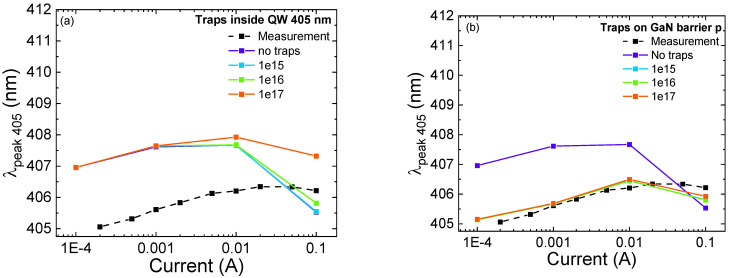
Peak wavelength variation as a function of current for the 405 nm QW. The black dashed line represents the experimental results, whereas the colored curves are the simulation results for different trap concentrations. (**a**) shows the effect of traps located inside the 405 nm QW; (**b**) shows the effect of traps located inside QB1.

**Figure 10:**
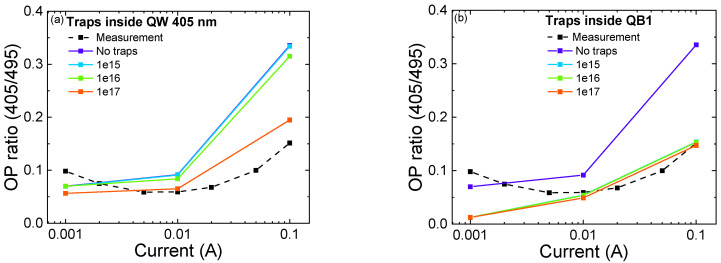
Effect of different trap concentrations on the ratio of the OP emitted by two quantum wells. The dashed line represents the experimental results. (**a**) shows the effect of traps located inside the 405 nm QW; (**b**) shows the effect of traps located inside QB1.

We obtained good matches for the trend as a function of current of the peak wavelength of the 405 nm QW (Figure 9a,b), and the optical power ratio (Figure 10a,b). In a high-current regime, we can observe that, by adding traps inside the 405 nm QW and QB1, close to the p-side, we are able to reproduce the experimental results. To be more specific, the increase in trap concentration in the 405 nm QW leads to a reduction in the blue-shift (of the 495 nm well) at high current, suggesting a decrease in the concentration of carriers in the QW, and a consequently lower screening of the QCSE [6,9,35]. Figure 10a,b illustrate the comparison between the experimental optical power ratio and the simulated one as a function of different trap concentrations inside the 405 nm QW and QB1, respectively. We were able to reproduce the trend at a high injection regime with the increment of trap concentration inside the QB1 and 405 nm QW.

In particular, the increment of traps causes a decrease in the optical power ratio (Figure 10) due to the optical power decay of 405 nm QW. This could be explained by an enhancement of defects that act as NRRCs [5,36]. Moreover, the optical power of the 495 nm QW is affected by the presence of traps in QB1 and the 405 nm QW only for high trap densities (*N_t_sim_* = 10^17^ cm^−^^3^) [16,37]. The non-optimized matching for low injection currents could be related to the presence of carrier injection mechanisms assisted by defects, which are not implemented in our simulation. Such processes could favor electron injection inside the 405 nm QW, leading to better matching between experimental and simulated optical power ratios at low currents [38].

The analysis of the impact of spatial position on the simulated optical characteristics suggests that, in order to model the experimental results, it is necessary to consider the combined effect of defects located within the entire active region [28,29]. Therefore, our starting point for modeling the devices is to consider a trap density equal to *N_t_sim_* = 1 × 10^15^ cm^−^^3^, located at the midgap, with σ = 1 × 10^−15^ cm^2^, inside the two QWs and inside QB1 and QB3.

### 3.2. Optical Degradation

**Figure 11:**
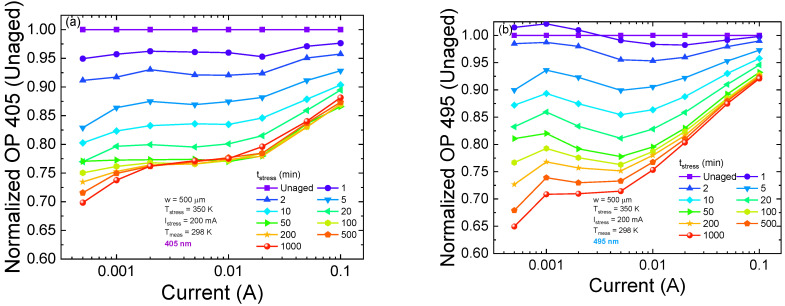

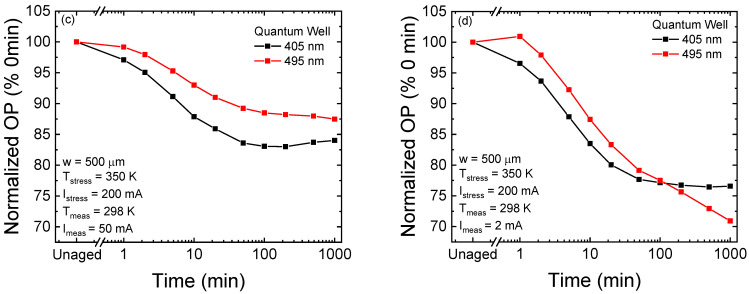
Optical power emitted by the 405 nm QW and 495 nm QW, normalized by the curve measured for the unaged device. (**a**) is the normalized L-I for the 405 nm QW, (**b**) refers to the 495 nm QW, (**c**) shows the optical degradation kinetics for a high injection current (I = 50 mA), and (**d**) refers to a low injection current (I = 2 mA).

In this section, we present the results obtained during a stress test carried out for 1000 min at high temperature (T = 350 K), with a constant current stress at I = 200 mA. During stress, the optical characteristics were monitored at different time steps at room temperature. In order to identify the device layers where degradation preferentially occurs, we separately analyzed the optical emission of the two quantum wells. Figure 11a,b show the optical power emitted by the 405 nm QW and 495 nm QW, respectively, normalized by the curve measured for the unaged device. We can see (Figure 11c,d) that degradation occurs in two phases, with different degradation rates for the two quantum wells. In particular, during the first phase, lasting up to 50 min of stress, optical degradation is stronger for the 405 nm QW for every injection condition. During the second phase, optical degradation slows down in the low-injection regime, whereas a slight recovery (1%) is observable for high injection currents. The trend suggests that the optical degradation mechanisms affecting the device characteristics in this regime reach saturation after the onset of the second phase of stress.

**Figure 12:**
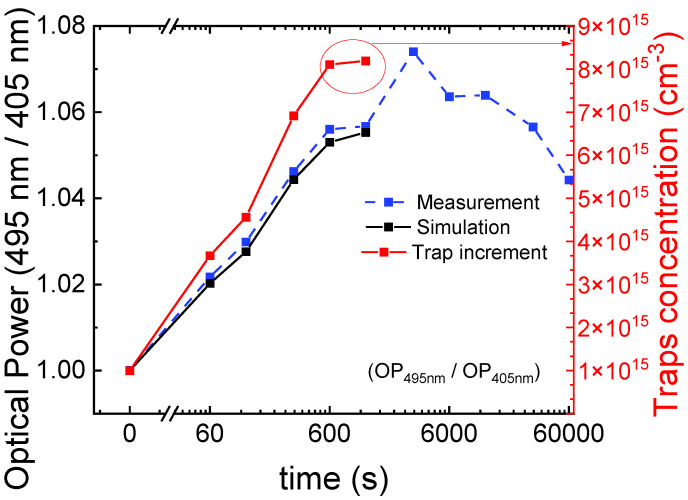
Optical power ratio, calculated as the optical power emitted by the 495 nm QW divided by the emission of the 405 nm QW, measured during stress. The right axis shows the corresponding estimated increment in trap density inside the 405 nm QW as a function of the time required to obtain the same trend in the experimental data.

**Figure 13:**
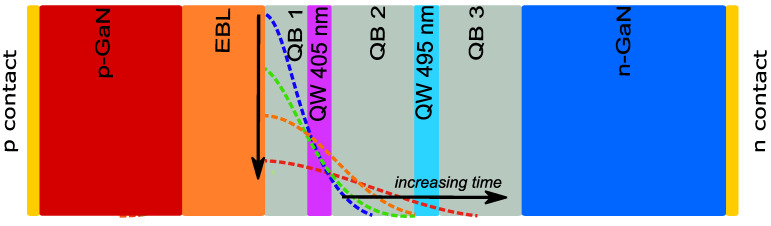
Scheme of the diffusive front.

Simulations (Figure 12) were carried out considering the condition of defects located in QB1, QB3 and the QWs, with a density *N_t_sim_* = 1 × 10^15^ cm^−^^3^. We were able to model the degradation, up to 50 min of stress, as being due to an increment in the concentration of traps inside the 405 nm QW. This result suggests that traps located inside this specific QW cause optical degradation associated with phase 1. Simulation results support the hypothesis of a diffusive front that originates from the p-side and moves towards the n-side (Figure 13) [39,40]. Therefore, during the first phase, the diffusive front leads to an increment of defectiveness inside the 405 nm QW, which is closer to the p-doped EBL, which causes an optical power decay due to the enhancement of SRH recombination. With the progression of stress, the front moves towards the n-side, so the degradation mechanism reaches saturation for the 405 nm QW. Moreover, simulations show that the impact of the trap increment (from 1 × 15 cm^−^^3^ to 1 × 17 cm^−^^3^) within the 405 nm well or within QB1 has a stronger effect for a high injection level. In particular, for a low-current level, the presence of traps could probably modify the band bending, improving the injection efficiency, thus compensating for the increment of the SRH rate.

**Figure 14:**
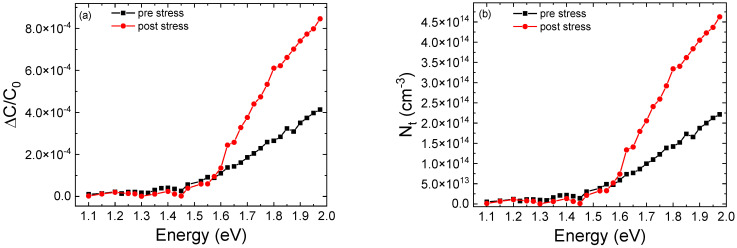
$\;\left(a\right)\;\frac{\Delta C}{C_{0}}\;$measured before and after stress. (**b**) shows trap density calculated from the $\frac{\Delta C}{C_{0}}$ measurement according to Equation (7). There is an increment of the trap density during the aging process as we also observed in simulations.

The overall increment of defectiveness due to the aging process is confirmed by DLOS measurements. As shown in Figure 14, we can observe an increase in $\frac{\Delta C}{C}$ for the stressed device. According to the following approximated formula [11]:

(7) $$N_{t\_meas}≅\;2\cdot \frac{\Delta C}{C_{0}}\cdot N_{D}$$

where *N_T_meas_* is the trap density, *N_D_* is the charge density in the active region extrapolated from capacitance-voltage measurement, and $\frac{\Delta C}{C_{0}}$ was extracted from DLOS measurements, the increase in $\frac{\Delta C}{C}$ can be related to an increase in trap concentration. The lowest value of *N_t_meas_* is about 2 × 10^14^ cm^−^^3^ for the pre-stress condition.

Regarding the 495 nm QW (Figure 11b,d), one can see that degradation during the first phase is delayed with respect to the 405 nm QW, possibly due to the presence of a competing process that is opposing the degradation, or due to the larger distance from the source of defects. During the second phase of stress, we see that degradation is stronger for this QW, in particular in the low-injection regime. This process can be ascribed to an increment of SRH recombination due to the increase in trap concentration associated with the diffusive front, or due to a lowering of the injection efficiency, since both are compatible with a rebalancing of the rate equation and to the relocation of NRRCs, which may also electrostatically affect carrier injection. Moreover, in the low-bias regime, the stronger degradation of the 495 nm QW could also be related to Auger-driven defect generation processes [13,32] whose rates are higher in the 495 QW due the higher carrier concentration that this well exhibits during stress with respect to the other one, as demonstrated both by simulations and by the stronger OP emitted from the 495 nm QW [41].

## 4. Conclusions

In conclusion, in this work we studied the impact that the spatial location of deep levels associated with defects has on the optical performance of color-coded multi-quantum-well InGaN-based light-emitting diodes. To this end, we developed a physics-based simulation deck by means of the Apsys suite (by Crosslight). Preliminary characterization and modeling of the devices allowed us to explain the stronger emission of the 495 nm well as being due to the poor electron injection into the 405 nm well.

The analysis of the effects that different trap locations have on the optical performance suggests the hypothesis that the unaged device has defects within the active region. As indicated by DLOS measurements, when the device is submitted to stress testing, an increment in the concentration of the pre-existent defects occurs. As those defects are located around the midgap, representing the worst-case for the non-radiative recombination, this increase is assumed to induce the observed optical decay. The degradation rate is different for the two quantum wells, and occurs in two phases. The first phase mainly regards the 405 nm QW, and follows an exponential trend, suggesting that the mechanism reaches saturation. This process was successfully modeled by considering an increment of traps inside the 405 nm QW [10,36,42]. We suggest the presence of a diffusive front of NRRCs, which moves from the p-side towards the n-side. During the second phase of stress, for high injection levels, the optical power decay for the 495 nm QW can be ascribed to an increment of SRH recombination due to the increment of defects that act as NRRCs, or to a lowering of the injection efficiency. However, for low-measuring-current levels, during both phases, degradation is stronger for the 495 nm QW, possibly due to the higher carrier density that favors the generation of defects through recombination-enhanced events [41].
